# The Host Response to Viral Infections Reveals Common and Virus-Specific Signatures in the Peripheral Blood

**DOI:** 10.3389/fimmu.2021.741837

**Published:** 2021-10-27

**Authors:** Ephraim L. Tsalik, Cassandra Fiorino, Ammara Aqeel, Yiling Liu, Ricardo Henao, Emily R. Ko, Thomas W. Burke, Megan E. Reller, Champica K. Bodinayake, Ajith Nagahawatte, Wasantha K. Arachchi, Vasantha Devasiri, Ruvini Kurukulasooriya, Micah T. McClain, Christopher W. Woods, Geoffrey S. Ginsburg, L. Gayani Tillekeratne, Klaus Schughart

**Affiliations:** ^1^Duke Center for Applied Genomics and Precision Medicine, Duke University School of Medicine, Durham, NC, United States; ^2^Emergency Department Service, Durham Veterans Affairs Health Care System, Durham, NC, United States; ^3^Division of Infectious Diseases, Department of Medicine, Duke University School of Medicine, Durham, NC, United States; ^4^Duke Molecular Genetics and Microbiology, Duke University School of Medicine, Durham, NC, United States; ^5^Department of Electrical and Computer Engineering, Duke University, Durham, NC, United States; ^6^Department of Medicine, Duke Regional Hospital, Durham, NC, United States; ^7^Faculty of Medicine, University of Ruhuna, Galle, Sri Lanka; ^8^Teaching Hospital Karapitiya, Galle, Sri Lanka; ^9^Medical Service, Durham Veterans Affairs Health Care System, Durham, NC, United States; ^10^Department of Infection Genetics, Helmholtz Centre for Infection Research, Braunschweig, Germany; ^11^University of Veterinary Medicine Hannover, Hannover, Germany; ^12^Department of Microbiology, Immunology and Biochemistry, University of Tennessee Health Science Center, Memphis, TN, United States

**Keywords:** viral respiratory infections, host response (HR), human patients, influenza, enterovirus, rhinovirus (RV), metapneumovirus, dengue virus (DEN)

## Abstract

Viruses cause a wide spectrum of clinical disease, the majority being acute respiratory infections (ARI). In most cases, ARI symptoms are similar for different viruses although severity can be variable. The objective of this study was to understand the shared and unique elements of the host transcriptional response to different viral pathogens. We identified 162 subjects in the US and Sri Lanka with infections due to influenza, enterovirus/rhinovirus, human metapneumovirus, dengue virus, cytomegalovirus, Epstein Barr Virus, or adenovirus. Our dataset allowed us to identify common pathways at the molecular level as well as virus-specific differences in the host immune response. Conserved elements of the host response to these viral infections highlighted the importance of interferon pathway activation. However, the magnitude of the responses varied between pathogens. We also identified virus-specific responses to influenza, enterovirus/rhinovirus, and dengue infections. Influenza-specific differentially expressed genes (DEG) revealed up-regulation of pathways related to viral defense and down-regulation of pathways related to T cell and neutrophil responses. Functional analysis of entero/rhinovirus-specific DEGs revealed up-regulation of pathways for neutrophil activation, negative regulation of immune response, and p38MAPK cascade and down-regulation of virus defenses and complement activation. Functional analysis of dengue-specific up-regulated DEGs showed enrichment of pathways for DNA replication and cell division whereas down-regulated DEGs were mainly associated with erythrocyte and myeloid cell homeostasis, reactive oxygen and peroxide metabolic processes. In conclusion, our study will contribute to a better understanding of molecular mechanisms to viral infections in humans and the identification of biomarkers to distinguish different types of viral infections.

## Introduction

Respiratory viral infections represent a significant threat to human health worldwide. Prior to the SARS-CoV-2 pandemic, influenza was the most common cause of morbidity and mortality in adults with respiratory viral infections (1). Implementation of molecular diagnostics has demonstrated that other viruses such as metapneumovirus and rhinoviruses are also significant contributors to the overall burden of respiratory infections (2, 3). The host response to respiratory infection has been described in both experimental model systems and in human patients. In humans, transcriptome analyses have been performed to describe the response in the peripheral blood for several viral infections including influenza virus [e.g. (4–20)]. or SARS-CoV-2 [e.g. (21–28)]. Several studies described the specific transcriptome response in peripheral blood cells that distinguish viral from bacterial infections (6, 7, 9, 10, 29) or moderate from severe influenza infections (12, 14, 17, 18). However, few studies have distinguished transcriptomic responses to different viral pathogens (8, 23, 30, 31).

Here, we performed transcriptomic analysis from the peripheral blood of patients infected with different viruses including respiratory and non-respiratory infections. We identified conserved elements of the host response common to all included viruses and we identified virus-specific responses. These findings confirm the potential for host response diagnostics to differentiate between viral infections despite their shared clinical features.

## Materials and Methods

### Clinical Enrollment and Case Definitions

Patients were enrolled prospectively for sample collection in the Emergency Departments (EDs) of three hospitals from 2009 through 2016: Duke University Medical Center (Durham, NC), Durham VA Health Care System (Durham, NC), and UNC Health Care (Chapel Hill, NC). Enrollment was by convenience sampling as part of two consecutively executed observational studies: Community Acquired Pneumonia and Sepsis Study (CAPSS), and Rapid Diagnostics in Categorizing Acute Lung Infection (RADICAL) study. Patients were eligible for CAPSS if they were ≥6-years old with a known or suspected infection of <28-days duration and if they exhibited two or more systemic inflammatory response syndrome criteria (32). RADICAL enrolled patients age ≥2-years with acute respiratory illness of <28-days duration. Healthy controls were adults recruited as part of a longitudinal study evaluating community-onset respiratory viral infections among Duke University undergraduates. Samples were selected from asymptomatic participants (33). In Sri Lanka, consecutive patients ≥15 years of age who were hospitalized in the largest tertiary care hospital in the Southern Province were enrolled from June 2012 to October 2014. Subjects were eligible for enrollment within the first 48 hours of admission if they had documented fever ≥38°C and lacked signs of a focal bacterial infection (e.g., urinary tract infection). Demographic and clinical data were obtained at enrollment and during the course of hospitalization by interview and chart review. All subjects were noted to be of Sri Lankan descent. Acute dengue was confirmed using IgG ELISA, virus isolation, real-time reverse transcription polymerase chain reaction (RT-PCR) for DENV, and RT-PCR for flaviviruses, as previously described (34). Supplemental respiratory pathogen testing was performed for all subjects using either the ResPlex V2.0 (Qiagen; Hilden, Germany), Respiratory Viral Panel (Luminex; Austin, TX), or Respiratory Pathogen Panel (Luminex; Austin, TX). **Table S1** provides details of microbiological assay for individual study participants. Some assays did not distinguish between rhinovirus and enterovirus. Therefore, infections due to either virus were considered as one category. EBV and CMV can both be detected in the blood long after acute infection, making it difficult to determine whether their identification is infection or just a bystander. However, the cases included here were determined through clinical adjudication to represent true infection in the forms of mononucleosis and acute CMV infection. Clinical adjudications were performed to identify the microbiological etiology of illness except for those enrolled in Sri Lanka (6). Adjudicators were clinicians experienced in managing patients with ARI, defined either by subspecialty training in a relevant field or by >2 years of post-graduate clinical experience in that field. Relevant areas of expertise included hospital medicine, emergency medicine, infectious diseases, or pulmonary/critical care medicine. Information available to adjudicators included the electronic medical record, supplemental etiology testing, and the case report. Adjudications were performed at least 28 days after enrollment and before any gene expression data were generated. All subjects included in this analysis had a microbiologically confirmed viral infection with a clinical syndrome compatible with a viral illness. Compatible respiratory viral clinical syndromes included upper respiratory infection, rhinosinusitis, pharyngitis, laryngitis, acute bronchitis, acute exacerbation of chronic obstructive pulmonary disease, bronchopneumonia, and pneumonia. No bacterial/viral co-infections were included in this analysis.

### Preparation and Sequencing of Blood RNA

Upon enrollment, whole blood was collected into PAXgene Blood RNA tubes (Qiagen) and processed according to manufacturer instructions with storage at -80°C. Total RNA was extracted using QIAsymphony PAXgene Blood RNA Kit on QIAsymphony SP instrument (QIAGEN). RNA yield was determined using Qubit RNA BR Assay Kit read on Victor X2 Plate Reader (Perkin Elmer), and quality assessed using Agilent HS RNA (15NT) Kit read on Fragment Analyzer System (Agilent). Strand-specific RNA sequencing libraries were generated using NuGEN Universal Plus mRNA-Seq kit with AnyDeplete-mediated human globin transcript depletion (NuGEN, Redwood City, CA). The libraries were sequenced on Illumina NovaSeq 6000 S4 flow cell at 150bp paired end reads with an average of 50M read pairs per RNA sample.

### Bioinformatic Analysis

Reads were quality checked with package FastQC (version 0.11.4, http://www.bioinformatics.babraham.ac.uk/projects/fastqc), then quality trimmed using Trimgalore (version 0.4.4, https://www.bioinformatics.babraham.ac.uk/projects/trim_galore/) with default settings. Trimmed reads were mapped to human genome annotation hg38 (ENSMBL hg38 release 91) using STAR [version 2.5.2b (35)] with default settings. Analysis and visualization of expression data was performed using the R software package (version 3.4.0) (36). Mapped reads were counted using RsubRead [version 1.32.4 (37)]. Raw counts from human genome were then normalized using DESeq2 [version 1.16.1 (38)]. Principal component analysis (PCA) was used to visualize groups (pathogens, sex) and identify outliers. One extreme outlier was removed based on the PCA. For identification of differentially expressed genes (DEG), the Limma package [version 3.42.2 (39, 40)] was used. DEGs were identified based on an adjusted p-value of < 0.05 and exhibiting more than a 1.5-fold (log2 = 0.5849625) difference in expression levels. Multiple testing adjusted p-value were calculated according to Benjamini and Hochberg (41). Volcano plots were generated with the package EnhancedVolcano, version 1.8.0 (42). Pathway association analysis was performed with the package clusterProfiler (43). Digital cell quantification was performed with package ComICS (44).

### Data Availability

Raw and processed data were deposited at the public GEO gene expression database (https://www.ncbi.nlm.nih.gov/geo/; GEO ID: GSE157240).

## Results

### Cohort Characteristics

Subjects were recruited from three U.S. EDs based on the presence of acute respiratory infection or suspected sepsis. Subjects were also enrolled in Sri Lanka based on the presence of acute, undifferentiated febrile illness. Only those confirmed to have viral etiologies were included in this analysis. The dataset consisted of 182 samples including 20 healthy controls. The group was well-balanced by gender with 98 females and 84 males. The mean age was 39 years with a range of 15 to 90 years. **Table 1** shows the demographic and clinical data for all participants. Our analysis focused primarily on 65 subjects with influenza A virus (IAV), 31 with enterovirus/rhinovirus (ENV), and 17 with human metapneumovirus (MPV). Additional comparator groups were comprised of 21 subjects with dengue (DENV) and 28 subjects with other viruses [10 parainfluenza (PIV), 7 respiratory syncytial virus (RSV), 5 adenovirus (ADV), 3 cytomegalovirus (CMV), and 3 Epstein-Barr virus (EBV)].

**Table 1:** Demographics and clinical characteristics of study participants.

		Dengue	Influenza	Enterovirus/Rhinovirus	Metapneumo-virus	Other VIruses*	Healthy Control	Total
n		21	65	31	17	28	20	182
Age								
	Mean	34.2	40.5	40.5	60.1	42.5	18.3	39.4
	Range	15-72	18-80	19-86	33-90	19-80	18-20	15-90
Gender								
	Male	14	27	12	8	12	11	84
	Female	7	38	19	9	16	9	98
Race								
	Black	1 (5.0%)	34 (52.3%)	19 (61.3%)	7 (41.2%)	13 (46.4%)	1 (5.0%)	75 (41.2%)
	White	1 (5.0%)	23 (35.4%)	7 (22.6%)	10 (58.8%)	13 (46.4%)	13 (65.0%)	67 (36.8%)
	Asian	19 (90.5%)	7 (10.8%)	4 (13.0%)	0 (0%)	1 (3.6%)	5 (25.0%)	36 (19.7%)
	Unknown/Other	0 (0%)	1 (1.5%)	1 (3.0%)	0 (0%)	1 (3.6%)	1 (5.0%)	4 (2.2%)
Fever		20 (95.2%)	64 (98.5%)	26 (83.9%)	16 (94.1%)	20 (71.4%)	0 (0%)	146 (80.2%)
Symptom Score**	Mean	3.4	5.8	5.3	5.6	4.5		5.2
	Median	4	6	5	6	5		5
	IQR	1 (3-4)	2 (5-7)	3 (4-7)	2 (5-7)	2.25 (3.75-5)		2 (4-6)
Comorbidities***								
	Chronic Lung Disease		9 (18.8%)	12 (38.7%)	7 (41.2%)	3 (10.7%)	0 (0%)	31 (17.0%)
	Hypertension		21 (32.3%)	8 (25.8%)	9 (52.9%)	10 (35.7%)	0 (0%)	49 (26.9%)
	Hyperlipidemia		11 (16.9%)	6 (19.4%)	7 (41.2%)	6 (21.4%)	0 (0%)	30 (16.4%)
	Smoking		21 (32.3%)	15 (48.4%)	6 (35.3%)	14 (50%)	0 (0%)	57 (31.3%)
	Diabetes mellitus		9 (18.8%)	2 (6.5%)	4 (23.5%)	4 (14.2%)	0 (0%)	20 (11.0%)
	Cancer		1 (1.5%)	0 (9%)	5 (29.4%)	4 (14.2%)	0 (0%)	10 (5.5%)
Outcomes								
	Hospital Admission	19 (90.5%)	17 (26.2%)	9 (29.0%)	12 (70.6%)	15 (53.6%)		72 (39.6%)
	Mean Hospital LOS (days)****	6.1	4.4	14.4	9.8	4.4		7.0

*Other viruses includes parainfluenza (10), Respiratory Syncytial Virus (7),adenovirus (5), Epstein Barr Virus (3), and cytomegalovirus (3).

**Symptom severity score based on 7 symptoms (nasal congestion, sneezing, cough, malaise, sore throat, fever, headache). One point was awarded for each symptom.

***Comorbidity data was not collected for viral subjects enrolled in Sri Lanka (19 dengue, 7 influenza, 4 entero/rhinovirus, 1 parainfluenza).

****Mean hospital length of stay calculation did not include healthy controls or subjects not admitted.

### Host Responses in IAV, ENV, and MPV Infections Compared to Healthy Controls

**Figure 1:**
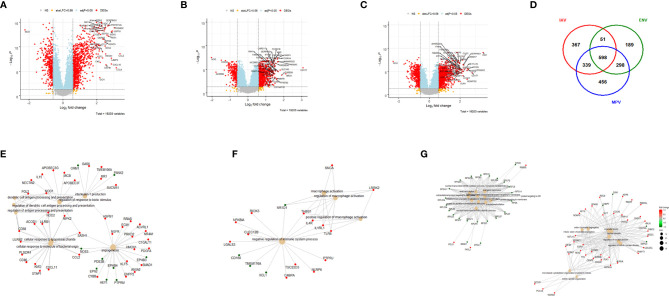
Host responses in IAV, ENV and MPV infections versus healthy controls. **(A)** Volcano plot of results of the contrast from the linear regression analysis of influenza virus infected versus healthy controls. y-axis: -log_10_ BH multiple testing adjusted p-values, x-axis: log_2_ fold change. DEGs (absolute log-fold change > 1.5, corresponding to a log_2_-fold change > 0.58; multiple testing adjusted p-value < 0.05) are colored red and the top 40 most strongly regulated (by log-fold change) DEGs are labeled. Blue: genes with adjusted p-value < 0.05. **(B)** Volcano plot of results of the contrast from the linear regression analysis of entero/rhino virus infected versus healthy controls. y-axis: -log_10_ BH multiple testing adjusted p-values, x-axis: log_2_ fold change. DEGs (absolute log-fold change > 1.5, corresponding to a log_2_-fold change > 0.58; multiple testing adjusted p-value < 0.05) are colored red and the top 40 most strongly regulated (by log-fold change) DEGs are labeled. Blue: genes with adjusted p-value < 0.05. **(C)** Volcano plot of results of the contrast from the linear regression analysis of MPV infected versus healthy controls. y-axis: -log_10_ BH multiple testing adjusted p-values, x-axis: log_2_ fold change. DEGs (absolute log-fold change > 1.5, corresponding to a log_2_-fold change > 0.58; multiple testing adjusted p-value < 0.05) are colored red and the top 40 most strongly regulated (by log-fold change) DEGs are labeled. Blue: genes with adjusted p-value < 0.05. **(D)** Venn diagram illustrating the overlaps between the DEGs from contrasts of IAV, ENV and MPV versus controls. A total of 2,298 DEGs were identified in all three infections (all genes combined part), 598 DEGs were commonly shared between the three infections (central part). IAV: DEGs from influenza versus healthy controls, ENV: DEGs from enterovirus/rhinovirus versus healthy controls, MPV: DEGs from human metapneumovirus versus healthy controls. **(E)** Functional analysis using GO term enrichment for the genes from the Venn diagram for IAV DEGs. Network of top 20 pathways and associated genes. Red: up-regulated, green: downregulated. **(F)** Functional analysis using GO term enrichment for the genes from the Venn diagram for ENV DEGs. Network of top 20 pathways and associated genes. Red: up-regulated, green: downregulated. **(G)** Functional analysis using GO term enrichment for the genes from the Venn diagram for MPV DEGs. Network of top 20 pathways and associated genes. Red: up-regulated, green: downregulated. NS, not significant.

We first compared the transcriptome responses for each respiratory virus (IAV, ENV, MPV) to healthy controls. **Table 2** lists the number of differentially expressed genes (DEGs) from these comparisons. Detailed results are provided in **Tables S2****–****S4**. The other respiratory viruses (ADV, CMV, EBV, PIV and RSV) were not included in these analyses because of inadequate small sample size. **Figures 1A–C** illustrate the DEGs for each virus *versus* healthy controls. The total number of DEGs were similar for each virus (1335, 1136 and 1691 for IAV, ENV and MPV, respectively). DEGs in the Venn diagram found only in one given virus infection were: 367 in IAV, 189 in ENV, and 456 in MPV-infected patients (**Figure 1D**). Functional analyses of the DEGs found only in one given virus were ‘interleukin-1 production’, ‘antigen processing’, ‘response to LPS and bacteria’ and ‘angiogenesis’ for IAV (**Figure 1E**); ‘negative regulation of immune system’ and ‘macrophage activation’ for ENV (**Figure 1F**); and ‘viral gene expression’, ‘protein targeting to ER and membrane’, and many pathways related to cell division for MPV-only DEGs responses when compared to healthy controls (**Figure 1G**).

**Table 2:** List of DEGs for comparison of single respiratory virus infections versus controls.

Comparison	Model	DEG total	DEG up	DEG down
IAV versus hlty	model.matrix(~ 0 + vir_grp2); IAV against healthy controls	1335	937	418
ENV/RHV versus hlty	model.matrix(~ 0 + vir_grp2); ENV against healthy controls	1136	815	321
MPV versus hlty	model.matrix(~ 0 + vir_grp2); MPV against healthy controls	1691	1110	581

Number of DEG for the different contrasts between patients with respiratory infections and healthy controls. Model, model used in limma for this comparison and groups included in model. DEG, differentially expressed genes; IAV, influenza; ENV/RHV, enterovirus/rhinovirus; MPV, metapneumovirus; hlty, healthy controls.

**Figure 2:**
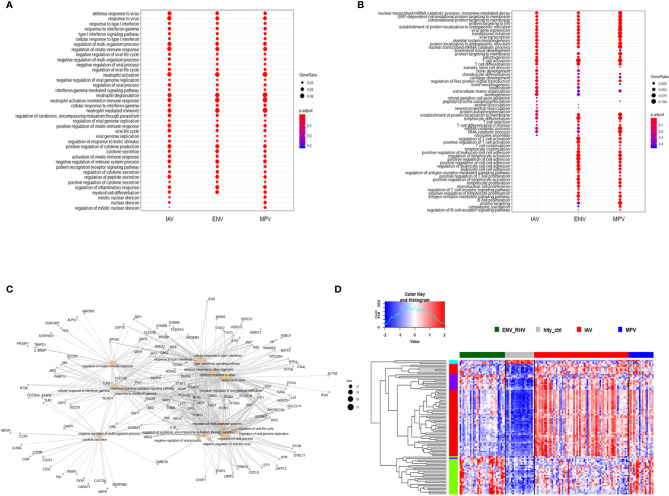
Functional analysis of DEGs. **(A)** Functional analysis using GO term enrichment for the combined set of all up-regulated DEGs from the comparison of IAV, ENV and MPV versus healthy controls. The top 30 (by p-value) pathways are presented. IAV, influenza virus; ENV, enterovirus/rhinovirus; MPV, metapneumovirus. **(B)** Functional analysis using GO term enrichment for the combined set of all down-regulated DEGs from the comparison of IAV, ENV and MPV versus healthy controls. The top 30 (by p-value) pathways are presented. **(C)** Functional analysis using GO term enrichment for the overlapping 598 DEGs (central part of ) of all three respiratory infections versus healthy controls. Note that all genes are grey since the fold-change cannot be displayed for the overlapping genes for three comparisons combined. **(D)** Expression levels of the top 150 DEGs from the comparisons of the three respiratory infection groups versus healthy controls (top 50 by log-fold change from each comparison) are presented. Values were scaled by row. red: up-regulated DEGs, blue: down-regulated DEGS. Note, that the combined 150 genes contained duplicates and the figure represents a total of 95 unique genes. IAV, influenza virus; ENV_RHV, enterovirus/rhinovirus; MPV, metapneumovirus; hlty_ctrl, healthy controls.

Furthermore, we combined all DEGs from the three comparisons to healthy controls resulting in 2,298 DEGs (all genes from **Figure 1D**). We then performed a comparative cluster pathway association analysis for all up-regulated DEGs and all down-regulated DEGs. The up-regulated DEGs were mainly associated with ‘response to virus, ‘interferon pathway’, ‘activation of innate immune response’, and ‘cytokine secretion’ (**Figure 2A**). The down-regulated DEG pathways were enriched for ‘protein targeting to membranes’, ‘cell differentiation’, and ‘T cell activation and differentiation’ (**Figure 2B**). There were 598 DEGs that overlapped between all comparisons (center of **Figure 1D**). The functional analysis of these overlapping DEGs showed a dominant representation of host responses to viral infections, ‘regulation of innate immune response’, ‘cellular response to type 1 interferon’, ‘response to virus’, ‘cytokine secretion’, ‘regulation of viral process’ (**Figure 2C**). To visualize these host responses, we selected the top 50 most differentially expressed genes (by absolute fold-change) from each virus-to-healthy comparison and represented them as a heatmap (**Figure 2D**). Most genes were up-regulated compared to healthy controls, and the largest group of up-regulated genes was observed for IAV suggesting that the response to IAV was strongest compared to the other two infections. The latter observation became more obvious when we investigated the magnitude of responses for the three pathogens as detailed below.

### Differences in the Magnitude of Host Responses

**Figure 3:**
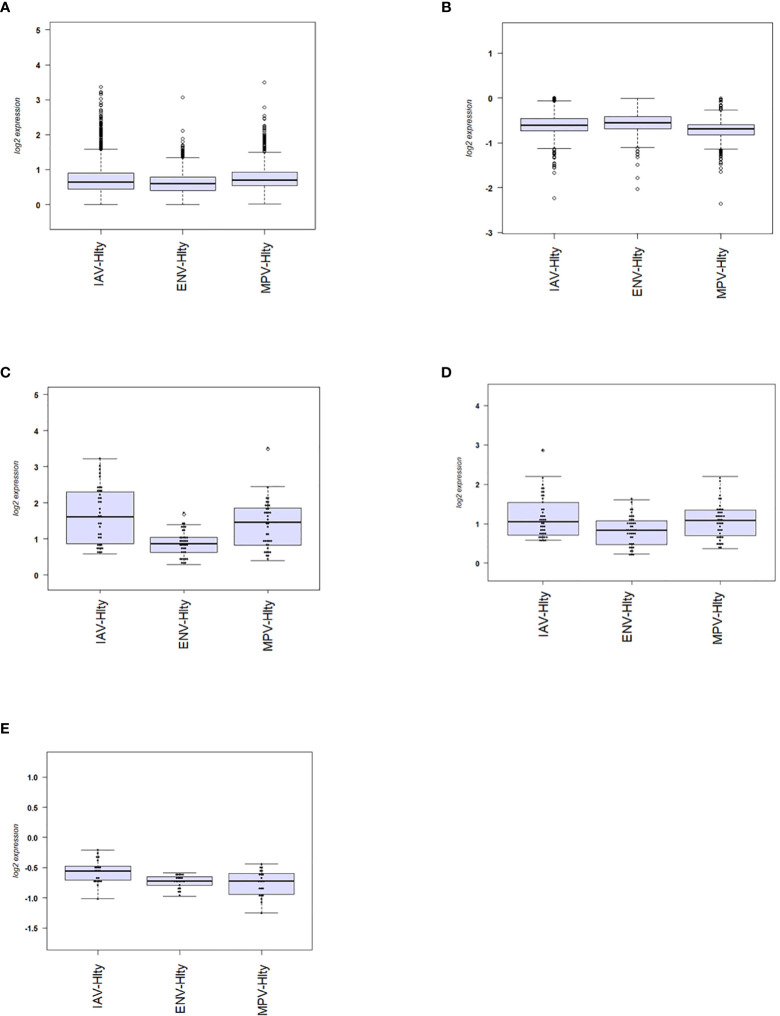
Magnitude of responses of DEG. **(A)** The mean fold change difference compared to healthy controls was calculated for up-regulated DEGs induced by each virus. Differences between IAV and ENV and between MPV and ENV were statistically significant. Y-axis: difference in log2 fold expression compared to healthy controls. **(B)** The mean fold change difference to healthy controls was calculated for each pathogen group and all down-regulated DEGs. Differences between MPV groups and ENV and between MPV and IAV groups were statistically significant. Y-axis: difference in log_2_ fold expression to healthy controls. **(C)** The mean fold change difference to healthy controls was calculated for each pathogen group and DEG associated with pathway ‘Response to type I interferon’. The differences between IAV and MPV groups and between MPV and ENV groups were statistically significant. Y-axis: difference in log_2_ fold expression compared to healthy controls. **(D)** The mean fold change difference to healthy controls was calculated for each pathogen group and DEG associated with pathway ‘Cytokine secretion’. The differences between IAV and ENV groups between ENV and MPV groups were statistically significant. Y-axis: difference in log_2_ fold expression to healthy controls. **(E)** The mean fold change difference to healthy controls was calculated for each pathogen group and DEG associated with pathway ‘Regulation of T cell activation’. The differences between IAV groups and MPV and between IAV and ENV groups were statistically significant. Y-axis: difference in log_2_ fold expression to healthy controls. IAV-Hlty: difference in normalized gene expression levels per gene between IAV infected and healthy controls; ENV-Hlty: difference in normalized gene expression levels per gene between ENV infected and healthy controls; MPV-Hlty: difference in normalized gene expression levels per gene between MPV infected and healthy controls.

We then investigated the magnitude of the host response to the three viruses by calculating the degree of differential gene expression relative to healthy controls (mean fold-change for the total 2,298 DEGs gene between infected and healthy group). For all up-regulated DEGs, the magnitude of differential expression was greatest for IAV and MPV (**Figure 3A**). For all down-regulated genes, the magnitude was greatest for MPV (**Figure 3B**). We also determined whether the magnitude of responses in selected pathways was different among the three respiratory viruses. ‘Response to type I interferon’ (**Figure 3C**) and ‘Cytokine secretion’ (**Figure 3D**) were the main response pathways to viral infections. They were most upregulated in the IAV group, followed by MPV, and then ENV. For the down-regulated pathways, ‘Regulation of T cell activation’, MPV had the most pronounced decrease in expression followed by ENV and then IAV (**Figure 3E**). Although the three viruses induced similar qualitative changes in gene expression and associated pathways, these results highlight that there are virus-specific differences in the magnitude of changes in infected versus control samples.

### Virus-Specific Responses in IAV and ENV Infections

To identify host responses that more specifically characterize a single viral infection compared to other respiratory viruses, we added gene expression data for subjects with adenovirus (ADV), cytomegalovirus (CMV), Epstein-Barr virus (EBV), parainfluenza virus (PIV), or respiratory syncytial virus (RSV) infections to our analysis and contrasted a single virus (IAV, ENV, or MPV) against all others in a linear regression model. Healthy controls were excluded. Virus-specific responses were not determined for the other respiratory viruses (ADV, CMV, EBV, PIV, RSV) due to low sample sizes. No MPV-specific response was found and so we only present data for the IAV- and ENV-specific responses.

**Figure 4:**
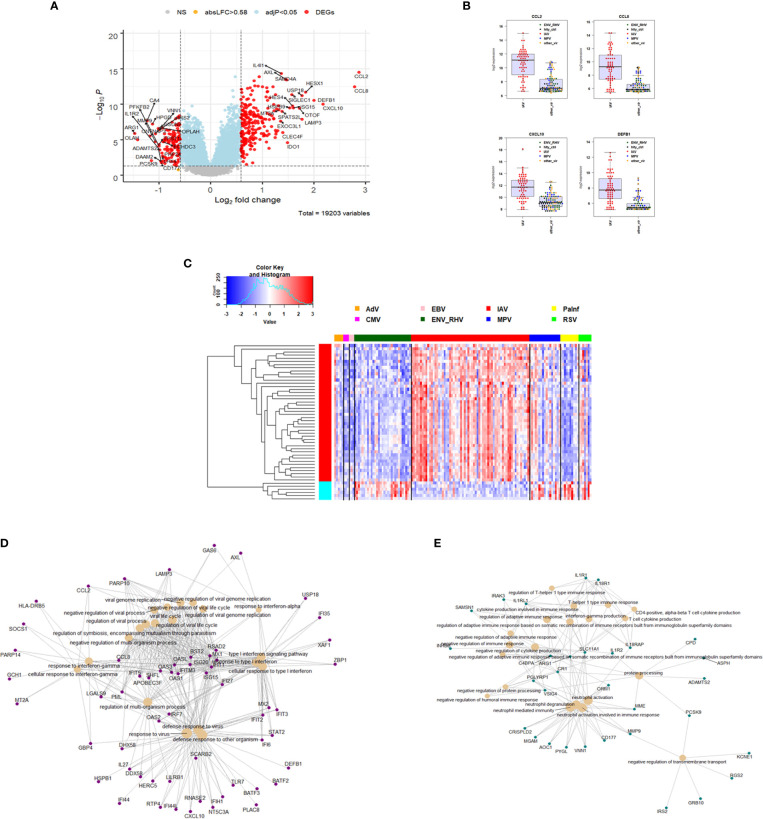
Responses in IAV versus other respiratory infections. **(A)** Volcano plot of results of the contrast from the linear regression analysis of influenza virus infected versus all other respiratory virus infections. y-axis: -log_10_ BH multiple testing adjusted p-values, x-axis: log_2_ fold change. DEGs (absolute log-fold change > 1.5, corresponding to a log_2_-fold change > 0.58; multiple testing adjusted p-value < 0.05) are colored red and the top 20 up- and down-regulated (by log-fold change) DEGs are labeled. Blue: genes with adjusted p-value < 0.05. **(B)** Normalized gene expression levels of the top four most strongly up-regulated (by absolute fold-change) influenza virus versus all other respiratory virus specific DEGs. *CCL2*, C-C Motif Chemokine Ligand 2; *CCL8*, C-C Motif Chemokine Ligand 8; *CXCL10*, C-X-C Motif Chemokine Ligand 10; *DEFB1*, Defensin Beta 1; IAV, influenza; ENV_RHV, enterovirus/rhinovirus; MPV, human metapneumovirus; other_vir, all other respiratory viruses; hlty_contr, healthy controls. **(C)** Heatmap of expression levels of the top 50 most strongly regulated (by absolute fold-change) DEGs from the contrast of IAV infected patients versus all other respiratory infections are presented. Values were scaled by row. red: up-regulated DEGs, blue: down-regulated DEGS. IAV, influenza virus; ENV_RHV, enterovirus/rhinovirus; MPV, metapneumovirus; PaInf, parainfluenza virus; RSV, respiratory syncytial virus; AdV, adenovirus; CMV, cytomegalovirus; EBV , Epstein-Barr virus. **(D)** Functional analysis using GO term enrichment for the DEGs from the contrast of IAV infected versus all other respiratory virus infections. Network of top 20 up-regulated pathways and associated genes. Red: up-regulated. **(E)** Functional analysis using GO term enrichment for the DEGs from the contrast of IAV infected versus all other respiratory virus infections. Network of top 20 down-regulated pathways and associated genes. Green: down-regulated. NS, not significant.

For identification of IAV-specific responses, we compared gene expression in 65 subjects with influenza against 97 subjects with non-IAV respiratory viral infections. This analysis revealed 374 DEGs (242 up- and 137 down-regulated genes, **Figure 4A**). The top four IAV up-regulated genes were *CCL2* (C-C Motif Chemokine Ligand 2), *CCL8* (C-C Motif Chemokine Ligand 8), *CXCL10* (C-X-C Motif Chemokine Ligand 10), and *DEFB1* (Defensin Beta 1) (**Figure 4B**). For details about their functions, see discussion. The complete list of DEGs can be found in **Table S5**. The heatmap for the top 50 most strongly (by absolute fold change) differentially regulated genes specific to IAV-infected patients showed a clear signature of up- and down-regulated genes that distinguished IAV from the other respiratory viruses (**Figure 4C**). Functional analysis of these IAV-specific DEGs revealed up-regulation of genes for pathways ‘response to interferon gamma, ‘response to interferon type I’, ‘regulation of viral process’, ‘defense response to virus’ (**Figure 4D**), and down-regulation of genes from pathways ‘T cell responses’, ‘neutrophil activation’, and ‘negative regulation of transmembrane transport’ (**Figure 4E**).

**Figure 5:**
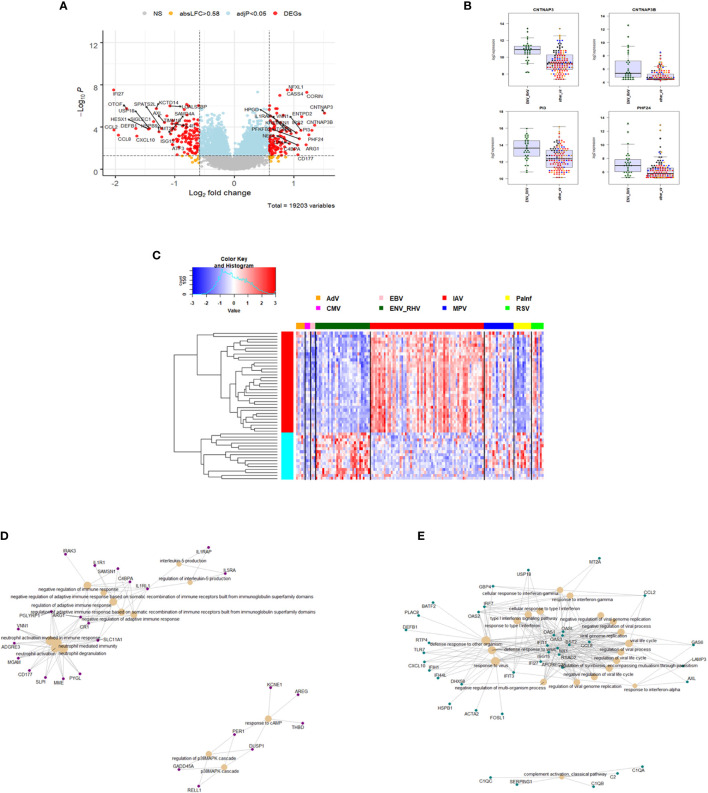
Responses in ENV versus other respiratory infections. **(A)** Volcano plot of results of the contrast from the linear regression analysis of ENV infected versus all other respiratory virus infections. y-axis: -log_10_ BH multiple testing adjusted p-values, x-axis: log_2_ fold change. DEGs (absolute log-fold change > 1.5, corresponding to a log_2_-fold change > 0.58; multiple testing adjusted p-value < 0.05) are colored red and the top 20 up- and down-regulated (by log-fold change) DEGs are labeled. Blue: genes with adjusted p-value < 0.05. **(B)** Gene expression levels of the top four most strongly up-regulated (by absolute fold-change) ENV versus all other respiratory virus specific DEGs. *CNTNAP3*, Contactin Associated Protein Family Member 3; *CNTNAP3B*, Contactin Associated Protein Family Member 3B; *PI3*, Peptidase Inhibitor 3; *PHF24*, PHD Finger Protein 24. IAV, influenza; ENV_RHV, enterovirus/rhinovirus; MPV, human metapneumovirus; other_vir, all other respiratory viruses; hlty_contr, healthy controls. **(C)** Expression levels of the top 50 most strongly regulated (by absolute fold-change) DEGs from the contrast of ENV infected patients versus all other respiratory infections are presented. Values were scaled by row. red: up-regulated DEGs, blue: down-regulated DEGS. IAV, influenza virus; ENV_RHV, enterovirus/rhinovirus; MPV, metapneumovirus; PaInf, parainfluenza virus; RSV, respiratory syncytial virus; AdV, adenovirus; CMV, cytomegalovirus; EBV , Epstein-Barr virus. **(D)** Functional analysis using GO term enrichment for the DEGs from the contrast of ENV infected versus all other respiratory virus infections. Network of top 20 up-regulated pathways and associated genes. Red: up-regulated. **(E)** Functional analysis using GO term enrichment for the DEGs from the contrast of ENV infected versus all other respiratory virus infections. Network of top 20 down-regulated pathways and associated genes. Green: down-regulated. NS, not significant.

We then compared the host response to ENV infections (31) to all other respiratory viruses (131). We identified 235 DEGs. Of these, 104 were up-regulated and 131 were down-regulated (**Figure 5A**). The top four up-regulated genes are shown in **Figure 5B**: *CNTNAP3*, *CNTNAP3B*, *PI3*, and *PHF24*. For details about their functions, see discussion. The complete list of DEGs can be found in **Table S6**. Of the 235 ENV-specific DEGs, 184 were also present in the IAV-specific signature. Although these 184 overlapping DEGs appeared in both ENV and IAV signatures, the magnitude or direction of change was different in the two viral infections. This is consistent with the observation that influenza induced the most robust host response while ENV induced the weakest host transcriptional response. Beyond the 184 overlapping DEGs in the EAV- and IAV-specific signatures, there were 51 DEGs appearing exclusively in the ENV signature and 190 exclusively in the IAV signature, which could serve as biomarkers to distinguish these viral infections. The heatmap of the top 50 most strongly regulated DEGs by absolute fold change (**Figure 5C**) revealed an ENV-specific pattern distinguishing these patients from other respiratory infections. Functional analysis of these ENV-specific DEGs revealed up-regulation of genes from pathways ‘neutrophil activation’, ‘interleukin 5 production’, ‘negative regulation of immune response’, ‘response to cAMP’, ‘p38MAPK cascade (**Figure 5D**), and down-regulation of genes from pathways ‘response to virus’, ‘response to interferon’, ‘regulation of viral process’, ‘complement activation, classical pathway’ (**Figure 5E**).

### Comparison of Respiratory With Non-Respiratory Viral Infections

**Figure 6:**
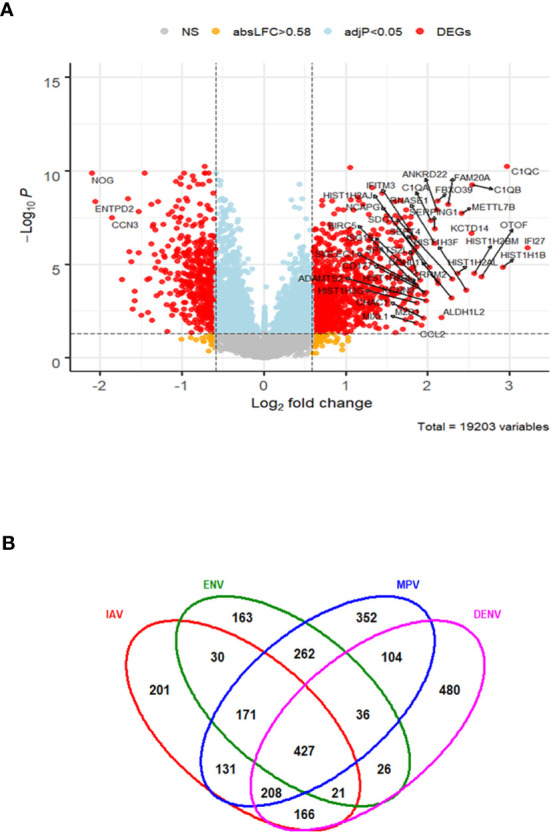
Host response in DENV infections. **(A)** Volcano plot of results of the contrast from the linear regression analysis of DENV infected versus healthy controls. y-axis: -log_10_ BH multiple testing adjusted p-values, x-axis: log_2_ fold change. DEGs (absolute log-fold change > 1.5, corresponding to a log_2_-fold change > 0.58; multiple testing adjusted p-value < 0.05) are colored red and the top 40 most strongly regulated (by log-fold change) DEGs are labeled. Blue: genes with adjusted p-value < 0.05. **(B)** Venn diagram of verlap of DEGs from single contrasts of respiratory and DENV infections to healthy controls. A total of 2778 DEGs were identified in all infections, 427 DEGs were commonly shared between all infections. 480 DEGs were only regulated in DENV infections. IAV: DEGs from influenza versus healthy controls, ENV: DEGs from entero/rhinovirus versus healthy controls, MPV: DEGs from metapneumovirus versus healthy controls, DENV: DEGs from dengue virus versus healthy controls. NS, not significant.

In order to characterize gene expression profiles in different types of viral infection, additional analyses included subjects infected with dengue virus (DENV)—a non-respiratory virus. We began by exploring the host response to DENV infection as compared to the healthy state. There were 1468 DEGs, including 941 up-regulated and 527 down-regulated genes (**Figure 6A**). The complete list of DEGs can be found in **Table S7**. The DEGs identified here exhibited a large overlap with the DEGs obtained from the comparisons of respiratory infections to healthy controls above. However, many DEGs were specific for DENV infections (480 of the total of 2,778 DEGs from all comparisons, **Figure 6B**).

**Figure 7:**
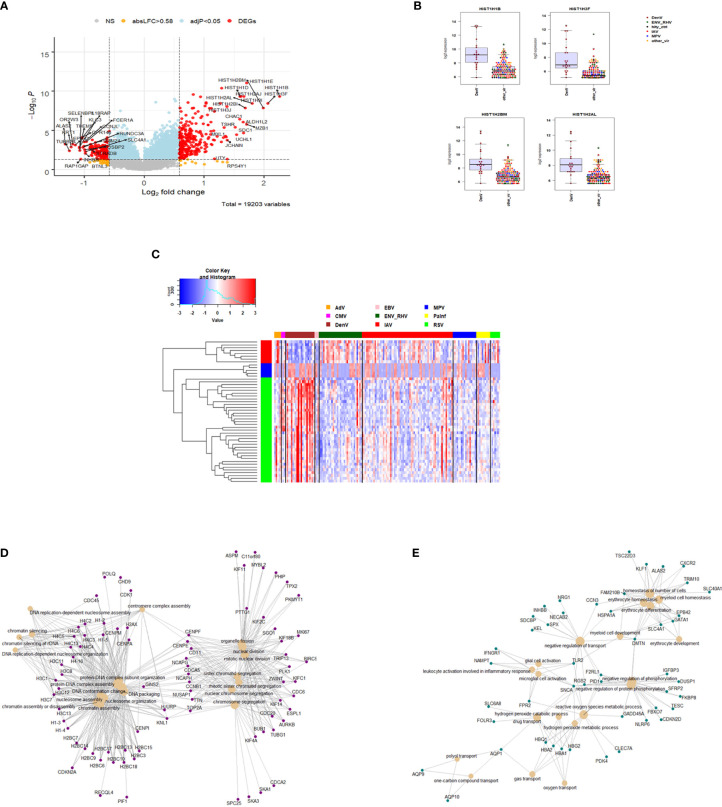
Responses in DENV versus other respiratory infections. **(A)** Volcano plot of results of the contrast from the linear regression analysis of DENV infected versus all other respiratory virus infections. y-axis: -log_10_ BH multiple testing adjusted p-values, x-axis: log_2_ fold change. DEGs (absolute log-fold change > 1.5, corresponding to a log_2_-fold change > 0.85; multiple testing adjusted p-value < 0.05) are colored red and the top 20 up- and down-regulated (by log-fold change) DEGs are labeled. Blue: genes with adjusted p-value < 0.05. **(B)** Gene expression levels of the top four most strongly up-regulated (by absolute fold-change) dengue virus specific DEGs versus all respiratory viruses. *HISTH1B*: H1.4 Linker Histone, Cluster Member, *HIST1H3F*: H3 Clustered Histone 7, *HIST1H2BM*: H2B Clustered Histone 14, *HIST1H2AL*: H2A Clustered Histone 16. IAV: influenza, ENV_RHV: enterovirus/rhinovirus, MPV: human metapneumovirus, DenV: dengue virus, other_vir: all other respiratory viruses, hlty_contr: healthy controls. **(C)** Heatmap of normalized expression levels of the top 50 most strongly regulated (by absolute fold-change) DEGs from the contrast of DENV infected patients versus all respiratory infections are presented. Values were scaled by row. red: up-regulated DEGs, blue: down-regulated DEGS. IAV, influenza virus; ENV_RHV, enterovirus/rhinovirus; MPV, metapneumovirus; PaInf, parainfluenza virus; RSV, respiratory syncytial virus; AdV, adenovirus; CMV, cytomegalovirus; EBV , Epstein-Barr virus; DENV, dengue virus. **(D)** Functional analysis using GO term enrichment for the DEGs from the contrast of DenV infected versus all other respiratory virus infections. Network of top 20 up-regulated pathways and associated genes. Red: up-regulated. **(E)** Functional analysis using GO term enrichment for the DEGs from the contrast of DENV infected versus all other respiratory virus infections. Network of top 20 down-regulated pathways and associated genes. Green: down-regulated. NS, not significant.

The host response to DENV infections has both shared and unique features to other viruses when compared to the healthy state. We next explored the DENV-specific host responses by comparing it to the response induced by other viral infections, not including healthy controls. This identified 429 DEGs specific for DENV infection (**Figure 7A** and **Table S8**). The top four DENV up-regulated genes are shown in **Figure 7B** as an example: *HISTH1B (*H1.4 Linker Histone, Cluster Member), *HIST1H3F (*H3 Clustered Histone 7), *HIST1H2BM (*H2B Clustered Histone 14), and *HIST1H2AL* (H2A Clustered Histone 16). The heatmap for the top 50 most strongly DEGs in DENV-infected subjects (**Figure 7C**) reveals virus-specific signatures. Functional analysis of the DENV-specific up-regulated DEGs showed enrichment of pathways for ‘DNA replication-dependent nucleosome assembly’, ‘nucleosome and chromatin assembly’, ‘chromosome segregation’, and ‘nuclear division’ suggesting a high level of cell proliferation (**Figure 7D**). Down-regulated DEGs were mainly associated with ‘erythrocyte and myeloid cell homeostasis’, ‘negative regulation of phosphorylation’, ‘reactive oxygen and peroxide metabolic processes’, and ‘oxygen transport’ (**Figure 7E**).

### Pan-Viral Response

**Figure 8:**
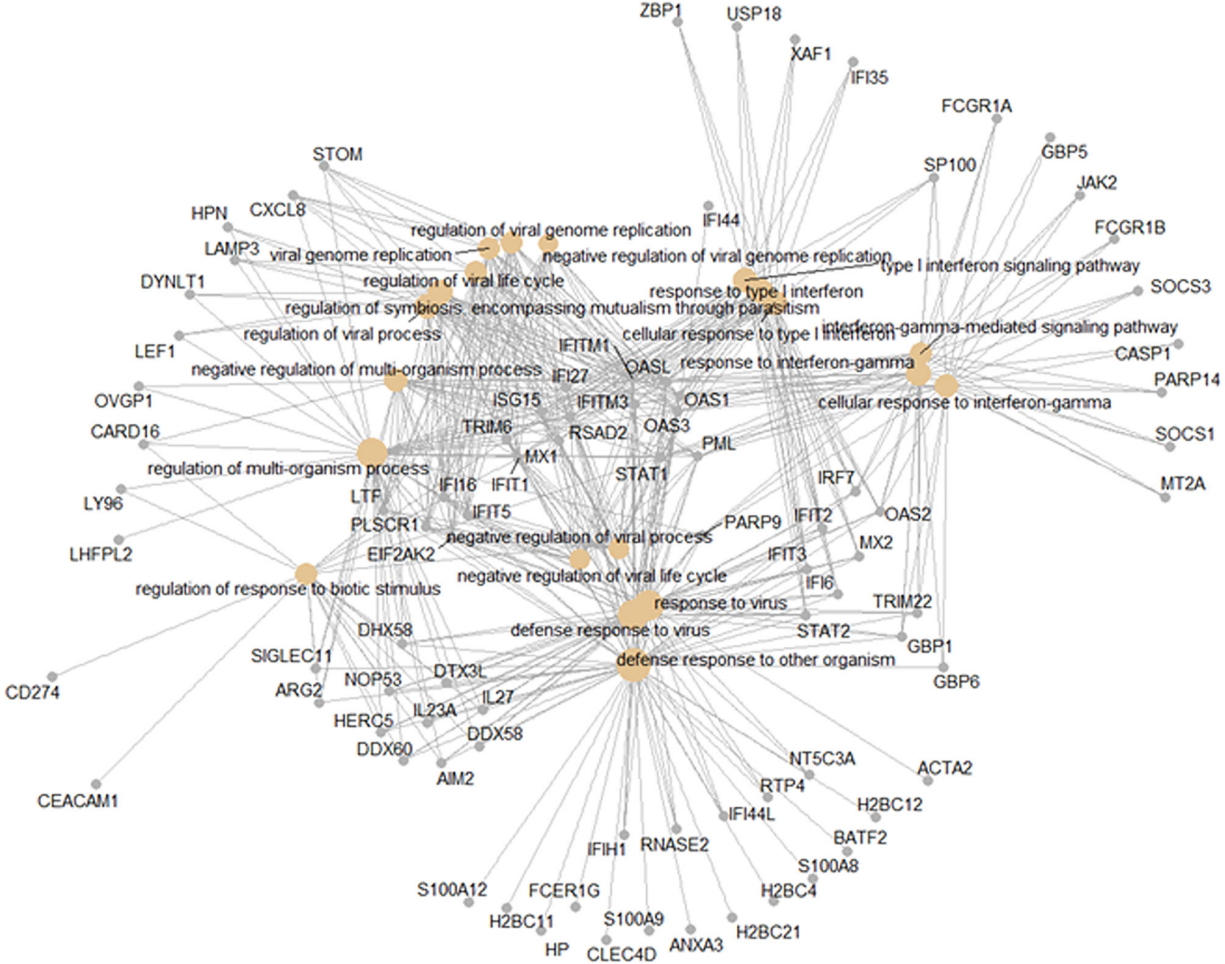
Pan-viral host response. Functional analysis using GO term enrichment for the overlapping 427 DEGs from the Venn diagram of the contrasts of all infections *versus* healthy controls. Network of top 20 pathways and associated genes.

The shared responses to dengue infection and other respiratory viruses could be considered a pan-viral response given the disparate clinical syndromes associated with these infections. As shown in **Figure 6B**, there were 427 DEGs common to all virus infections compared to healthy controls. Functional analysis of these pan-viral DEGs showed ‘response to virus’, ‘response to type I interferon’, and ‘response to interferon gamma’ pathways (**Figure 8**). Thus, activation of interferon pathways is a unifying aspect of the host response to viral infection.

### Digital Cell Quantification

**Figure 9:**
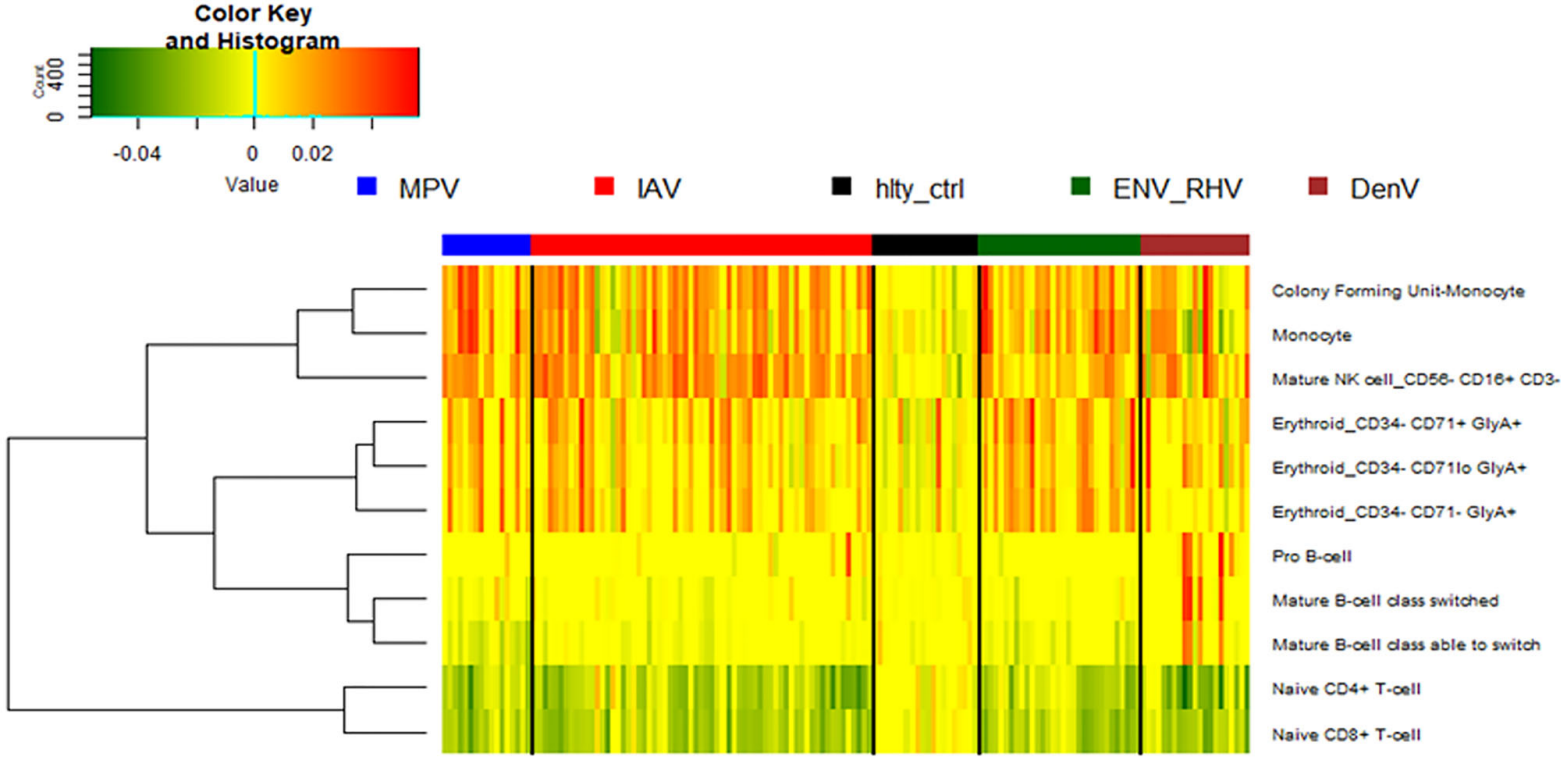
Digital cell quantification. Using digital cell quantification, immune cells were quantified based on gene expression levels. The relative abundance of immune cell populations is presented as a heatmap. Shown are only cell populations that exhibited differences across any infected/healthy group. IAV, influenza virus; ENV_RHV, enterovirus/rhinovirus; MPV, metapneumovirus; DenV, dengue virus.

We then performed a digital cell quantification, based on gene expression levels of all genes, to estimate the relative abundance of different cell populations in the different groups: IAV, ENV, MPV, and DENV infected subjects (**Figure 9**). Here, we observed an increase in monocytes, NK cells, and erythroblasts and a decrease in CD4 and CD8 T-cells in all infected patients. None of these responses were observed in controls. The only viral infection showing a unique pattern was DENV, which included an increase in B cells relative to the healthy state or other viral infections.

### Analysis of Confounding Factors

In addition to virus pathogen, several other confounding factors may contribute to the variation in gene expression. We therefore performed association analyses of virus pathogen, demographic and clinical confounders with the first ten principal components. This analysis revealed strong correlation of variation with viral pathogens, and in addition, with the occurrence of chronic lung diseases and with age categories young/adult/old (**Table S9**). The correlation with age categories was biased because most of the healthy controls were younger than 18 and most of the infected patients were older than 18. Sex, hypertension, hyperlipidemia, smoking, diabetes, and cancer did not show any strong correlations with the first principal components (**Table S9**). When analyzing only infected patients, the correlation with age category was weaker whereas the correlation with chronic lung disease was still significant (**Table S10**). To further explore the potential impact of chronic lung disease, we compared gene expression among influenza-infected subjects (the largest phenotypic group) with or without chronic lung disease. There was a similar number of DEGs whether or not chronic lung disease was present (1355 DEGs) or absent (1367 DEGs). Of these, 1329 DEGs overlapped between these two groups. In addition, we investigated the effect of hospitalization. Only IAV infected patients had reasonable sample sizes for a comparison (**Table S11**). However, when contrasting hospitalized *versus* non-hospitalized patients, we did not find any significantly differentially expressed genes.

## Discussion

Here, we studied the host response in the peripheral blood of patients infected with different viral pathogens. To our knowledge, this is the largest primary study of the host transcriptomic response to viral pathogens that includes several pathogens: influenza A virus (IAV), entero/rhino virus (ENV), metapneumovirus (MPV), parainfluenza (PIV), respiratory syncytial virus (RSV), adenovirus (ADV), cytomegalovirus (CMV), and Epstein-Barr virus (EBV). Furthermore, the comparison of respiratory infections to patients infected with a non-respiratory virus, dengue virus (DENV), has not been reported in any previously published study. We identified differentially expressed genes that are common to these infections, and signatures that are unique for individual pathogens.

The responses to all three major respiratory virus infections (IAV, ENV, MPV) were dominated by the up-regulation of genes from the interferon pathway as well as chemokine/cytokine related responses. This was the case for each single viral infection and for the overlap of the DEGs from the three respiratory viral infections. These observations are consistent with many other studies in humans [e.g. (4–20)]. including SARS-CoV-2 infection [e.g. (21–28)] and a meta-analysis of publicly available data (31).

Among the down-regulated DEGs, all three respiratory viruses induced changes that were enriched for protein targeting to membranes and cell differentiation pathways. However, ENV and MPV infections exhibited a stronger down-regulation of T cell activation and cell adhesion molecules compared to IAV infections. Down-regulation of adaptive immune response signals is a common feature of respiratory virus infections that has been described for SARS-CoV-2 (23, 26, 28, 45, 46) and for severe versus moderate IAV infections (12, 17). We speculate this pattern of immune response gene down-regulation in ENV and MPV infected subjects is consistent with the induction of a generally weaker host response. This hypothesis was further supported by the relative magnitude of host gene expression response, which was greatest for IAV, intermediate for MPV, and weakest for ENV, consistent with the severity of clinical disease associated with these infections (47–50). Alternatively, ENV and MPV may exert a stronger suppression of the host response by expressing viral genes that specifically down-regulate the host viral detection pathways compared to IAV, and therefore, innate immune signals are weaker.

The peripheral blood transcriptomic response to MPV infection has not yet been well described in the literature. It has been speculated that MPV more strongly suppresses the host interferon response than RSV infections (51). The comparison of host responses to MPV with RSV infections in animal models showed that MPV was a stronger inducer of interferon-α and interferon-γ (52). MPV inhibits pattern recognition-dependent signaling (RIG-I-like receptors) *in vivo*, resulting in reduced interferon induction and pathway activation (53, 54). BALB/c mice infected with human MPV had lower levels of inflammatory cytokines compared to RSV infections but a more sustained production of CXC cytokines (52, 54). Thus, these reports in the literature agree with our results showing that MPV induces an intermediate interferon response, lower than IAV but higher than ENV infections.

There is a growing interest in host response-based diagnostics, where most signatures focus on identifying the pathogen class (e.g., viral or bacterial) (55). Adding pathogen-specific host responses would augment the utility of such diagnostic approaches. This would be particularly relevant when new strains emerge where pathogen-detection tests are not readily available (e.g., influenza H1N1pdm09 and SARS-CoV-2).

Comparing the host response to IAV infections versus all other respiratory viruses, we identified 374 DEGs. The top four IAV up-regulated genes were *CCL2* (C-C Motif Chemokine Ligand 2), *CCL8* (C-C Motif Chemokine Ligand 8), *CXCL10* (C-X-C Motif Chemokine Ligand 10), and *DEFB1* (Defensin Beta 1). The first three genes represent cytokine/chemokine genes, which are a central component of the innate immune response. *DEFB1* is a member of the defensin gene family that consist of microbicidal and cytotoxic peptides mainly produced by neutrophils. The most down-regulated genes were *ARG1* (Arginase 1), *OLAH* (Oleoyl-ACP Hydrolase), *CA4* (Carbonic Anhydrase 4) and *CNTNAP3* (Contactin Associated Protein Family Member 3). *ARG1* catalyzes the hydrolysis of arginine to ornithine and urea. Arginine metabolism is a critical regulator of innate and adaptive immune responses where it is an antimicrobial effector pathway in polymorphonuclear granulocytes. *OLAH* contributes to the release of free fatty acids from fatty acid synthase, and *CA4* catalyze the reversible hydration of carbon dioxide. Both genes have no known function in the host defense against viral infections. *CNTNAP3* represents a cell-recognition molecule mediating glial cell contacts. It has not previously been implicated in in the host response to viral infections.

We also identified 235 ENV-specific DEGs. The top four up-regulated genes were *CNTNAP3*, *CNTNAP3B*, *PI3*, and *PHF24*. Note that *CNTNAP3* was downregulated in the IAV-specific response but was up-regulated in ENV infections. *CNTNAP3B*, like *CNTNAP3*, is a cell-recognition molecule mediating glial cell contacts and has not been identified as part of the host response to viral infection. *PI3* (PHD Finger Protein 24) has no known function and *PHF4* (Peptidase Inhibitor 3) represents a neutrophil-specific elastase with anti-microbial functions.

In contrast to IAV and ENV, we did not identify a MPV-specific signature. One explanation may be that the response to MPV is intermediate to the comparator viruses and therefore, the magnitude of difference between MPV and either IAV or ENV is too small to detect. Alternatively, there may have been too few MPV cases to detect small but significant differences. Future studies with more balanced and larger groups or a meta-analysis including other cohorts may resolve this issue.

Our results are in good agreement with similar studies described in the literature. A dataset published by Zhai et al. (8) reported transcriptomic data from adult subjects (127 – 131 depending on the sampling day) infected with different respiratory viruses and samples from the same individuals before infection. Of the 117 unique DEGs they reported as distinguishing viral infections from healthy controls, 108 overlapped with the viral vs. healthy comparison in our study. Thus, our results are well in agreement with this study. Andres-Terre et al. (31) performed a meta-analysis on published data for three respiratory viral infections (IAV, RSV, and rhinovirus) and identified an IAV signature including 127 genes. We compared this gene list with our IAV-specific list of 374 DEGs and found an overlap of 52 genes. The same authors also defined a smaller, 11-gene, IAV-specific gene list. Our IAV-specific list contained 10 of these 11 IAV-specific genes. In addition, they determined a common viral signature (‘meta-virus signature’) consisting of 396 genes. Our combined respiratory viral DEGs (total of 2,298 genes for all respiratory viruses combined) overlapped with this list for 115 genes. Although there are some genes included in each signature that do not appear on the other, the results are consistent particularly when focusing on the most discriminating DEGs. Abbasi et al. (30) reported that after infection with rhinovirus in pediatric patients, *CXCL10*, *CMPK2*, *RSAD2*, *SERPINA3*, and TNFAIP6 were up-regulated whereas *CXCL14*, *IVNS1ABP*, and *ZMAT3* were down-regulated in comparison to IAV infections. Similar to these findings, in our dataset, *IVNS1ABP* and *ZMAT3* were downregulated in ENV infections compared to IAV infection. In contrast, in our dataset, *CXCL10*, *CMPK2*, *RSAD2*, and *TNFAIP6* were significantly down-regulated in ENV infections compared to IAV infections. Thus, the direction of the regulation of genes in adult and pediatric patients may be different for several innate immune response genes. This hypothesis will have to be confirmed in future studies.

The host response to dengue infection overlapped considerably with that of other viruses. However, there were also notable DENV-specific responses including an up-regulation of histone-associated and cell cycle regulating genes. An analysis of protein-protein-interaction networks for these DENV-specific DEGs using the STRING interaction database, revealed networks regulated by the CDC6 (Cell Division Cycle 6) protein. CDC6 is essential for the initiation of DNA replication and functions as a regulator at the early steps of DNA replication. Almost all DENV-specific host response changes included an upregulation of gene expression. A notable exception was *BCL2L1 (*BCL2 Like 1). *BCL2L1* is involved in the regulation of mitochondrial membrane potential and controls production of reactive oxygen species and release of cytochrome C, which are potent inducers of cell apoptosis. Our observations suggest that DENV induces a greater degree of cellular proliferation of circulating immune cells than we observed for respiratory viral infections. This may be due to the systemic nature of dengue, which is not restricted to the respiratory tract. It is also possible that infection of blood lymphocytes by DENV itself initiates the proliferation of blood cells. The increase in pro-B cells, observed in our DCQ, may support this hypothesis. However, more target studies will be necessary to validate this interpretation. Future studies with single cell RNAseq will help address these possibilities. In addition, performing ATACseq on PBMCs of Dengue-infected subjects would be another important future approach to interrogate cell-specific chromatin changes.

Our study has several limitations. Most subjects in this study had mild or moderate clinical disease, limiting our ability to identify severity-dependent host changes. It is also possible that subjects with more severe infections, such as due to IAV or DENV, presented to clinical care earlier in the disease course. If so, it is possible that virus-specific differences could have represented different phases of the host response. There is also the possibility that confounding variables contributed to the observed gene expression differences. Most demographic variables were evenly distributed among the groups although this was not universally the case. Analyses to identify such confounding variables revealed age and chronic lung disease may have contributed. Although the viral pathogen itself was the largest contributor to observed differences, we cannot exclude the potential impact of these other variables. Information about duration of symptoms/time of sampling and the potential impact of treatment was not available for most subjects, precluding our ability to control for this. There was an age difference in the comparison groups, with infected being 40s to 60s, and healthy controls being more uniformly between 18-20. Moreover, the Sri Lanka subjects tended to be older than U.S. subjects. However, we did not adjust for age since there was still a significant amount of heterogeneity among ages within each group. We also note that nearly all subjects with DENV came from Sri Lanka, although the Sri Lanka cohort included several other types of infection as well. Nevertheless, we cannot discount the role that genetic variability may have played in these results. Here, bulk RNAseq was used to measure gene expression changes in the blood. Furthermore, analysis of respiratory samples may be more appropriate to study responses in infected tissues. This approach does not identify cell-specific expression changes. Future experiments could perform single cell RNAseq to confirm some of our findings and identify cell type-specific anti-viral host responses. Furthermore, information about the viral genomes (e.g., pathogen genome sequencing) could have identified pathogen-derived factors (e.g., variants) responsible for the observed host response differences. Another limitation is that gene expression analyses were performed in blood cells but not in the infected respiratory tract tissues, which may differ (56). Our study may contribute to the identification of novel biomarkers for different viral infections. However, it should be noted that more studies will be necessary to identify and validate clinically relevant biomarkers.

In conclusion, we compared the host response to different viral infections in humans and determined common pathways but also virus-specific differences in the host response at the molecular level. Inclusion of multiple types of viruses revealed there to be a pan-viral response. This study contributes to our understanding of the host transcriptional responses to viral infections in humans and the identification of biomarkers that could be used as a diagnostic strategy for pathogen identification. It provides a basis for follow-up validation studies in cell culture and animal models and identifies many genes and pathways as potential targets for future host-targeted drug development.

## Data Availability Statement

The datasets presented in this study can be found in online repositories. The names of the repository/repositories and accession number(s) can be found in the article text (M&M) and in the **Supplementary Material**.

## Ethics Statement

All studies involving human participants were reviewed and approved by the Duke University Institutional Review Board, the UNC Institutional Review Board, the Durham VA Institutional Review Board, and the Ethical Review Committee, Faculty of Medicine, University of Ruhuna. Written informed consent to participate in this study was provided by the participants’ legal guardian/next of kin.

## Author Contributions

ET, MM, CW, GG, and KS conceived and designed the experiments. ET, EK, CB, AN, WK, VD, RK, MM, CW, GT, and MR collected the samples. KS and TB performed the experiments. ET, AA, YL, RH, KS, and CF analyzed the data. ET, KS, and CF wrote the manuscript. ET, CW, MM, KS, and GG provided funding. All authors contributed to the article and approved the submitted version.

## Funding

This work was supported by intra-mural grants from the Helmholtz-Association (Program Infection and Immunity), a start-up grant from the University of Memphis Tennessee Health Science Center, NIAID Research Grants 2-U19-AI100625-06 REVISED and 5U19A|100625-07 awarded to KS. This work was also supported in part by the National Institute of Allergy and Infectious Diseases of the National Institute of Health [grant numbers U01AI066569 and UM1AI104681] and the U.S. DARPA [contract number N66001-09-C2082]. The content is solely the responsibility of the authors and does not represent the official views of the National Institutes of Health. CF was supported by the Eugene A. Stead Scholarship from Duke University School of Medicine. The funders had no role in study design, data collection and analysis, decision to publish, or preparation of the manuscript.

## Conflict of Interest

ELT, GSG, CWW, and TWB consult for and hold equity in Biomeme, Inc.

The remaining authors declare that the research was conducted in the absence of any commercial or financial relationships that could be construed as a potential conflict of interest.

## Publisher’s Note

All claims expressed in this article are solely those of the authors and do not necessarily represent those of their affiliated organizations, or those of the publisher, the editors and the reviewers. Any product that may be evaluated in this article, or claim that may be made by its manufacturer, is not guaranteed or endorsed by the publisher.
