# Supplementary material for: The Host Response to Viral Infections Reveals Common and Virus-Specific Signatures in the Peripheral Blood
Source: Front Immunol. 2021 Oct 27;12:741837. doi: 10.3389/fimmu.2021.741837 (PMC8578928; doi:10.3389/fimmu.2021.741837)
Supplement: Supplementary file 1 [file DataSheet_1.docx]

# Supplementary material

# The host response to viral infections reveals common and virus-specific signatures in the peripheral blood

Tsalik et al.

*** Correspondence:**Klaus Schughart
[kls@helmholtz-hzi.de](mailto:kls@helmholtz-hzi.de)

#### Table S1. Detailed list of diagnostic assay for each participant

File name: Table S1_110921.docx; Type of assay used for diagnosis of infection for each study participant.

#### Table S2. DEG list IAV versus control

File name: Table S2 ALL_limma_IAV_vs_hlty_ctrl_290121.csv; Description: table of differentially expressed genes from the comparison of IAV infected patients versus healthy controls. logFC: fold difference in normalized expression levels as log_2_; adj.P.Val: multiple testing adjusted P values.

#### Table S3. DEG list ENV versus control

File name: Table S3 ALL_limma_ENV_RHV_vs_hlty_ctrl_290121.csv; Description: table of differentially expressed genes from the comparison of ENV infected patients versus healthy controls. logFC: fold difference in normalized expression levels as log_2_; adj.P.Val: multiple testing adjusted P values.

#### Table S4. DEG list MPV versus control

File name: Table S4 ALL_limma_MPV_vs_hlty_ctrl_290121.csv; Description: table of differentially expressed genes from the comparison of MPV infected patients versus healthy controls. logFC: fold difference in normalized expression levels as log_2_; adj.P.Val: multiple testing adjusted P values.

#### Table S5. IAV versus all other respiratory viruses

File name: Table S5 limma_IAV_all_other_RV_290321.csv; Description: table of differentially expressed genes from the comparison of IAV infected patients versus all other respiratory virus infections. logFC: fold difference in normalized expression levels as log_2_; adj.P.Val: multiple testing adjusted P values.

#### Table S6 ENV versus all other respiratory viruses

File name: Table S6 limma_ENV_RHV_all_other_RV_290321.csv; Description: table of differentially expressed genes from the comparison of ENV infected patients versus all other respiratory virus infections. logFC: fold difference in normalized expression levels as log_2_; adj.P.Val: multiple testing adjusted P values.

#### Table S7 DENV versus healthy controls

File name: Table S7 ALL_limma_DenV_vs_hlty_ctrl_290121.csv; Description: table of differentially expressed genes from the comparison of DENV infected patients versus healthy controls. logFC: fold difference in normalized expression levels as log_2_; adj.P.Val: multiple testing adjusted P values.

#### Table S8: DEG list of DENV versus all other respiratory viruses

File name: Table S8 limma_DenV_all_other_RV_290321.csv; Description: table of differentially expressed genes from the comparison of DENV infected patients versus all other respiratory virus infections. logFC: fold difference in normalized expression levels as log_2_; adj.P.Val: multiple testing adjusted P values.

#### Table S9: Analysis of confounders


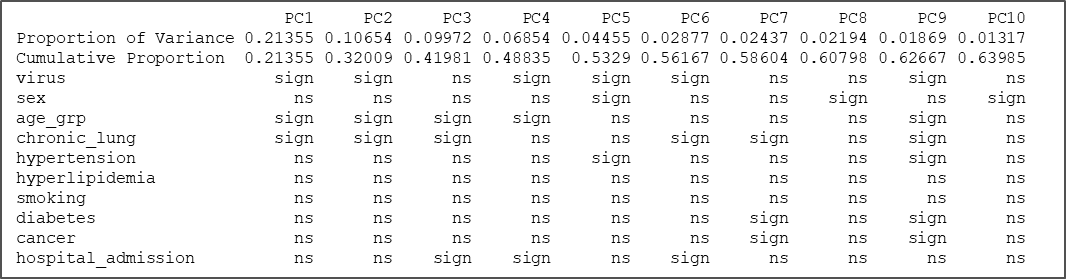


The first ten principal components of gene expression values from all groups were correlated by ANOVA with different clinical parameters of patients in the cohort. For virus pathogens, the following groups were used: DENV, ENV_RHV, healthy controls, IAV, MPV other_viruses (all other viruses combined). For the age groups the following categories were used: young (0 to 18), adult (18-65), old (>65).

#### Table S10: Analysis of confounders without healthy controls


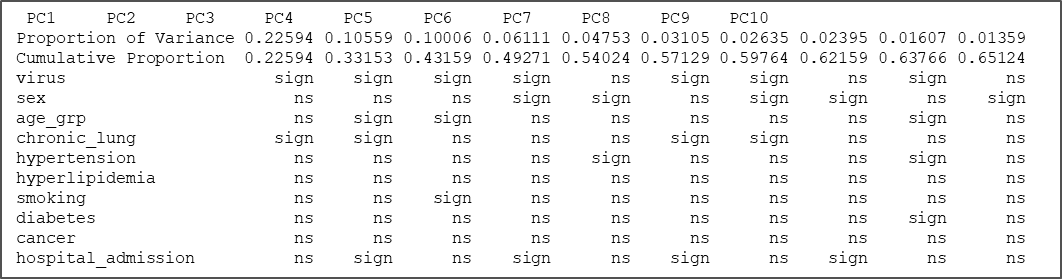


The first ten principal components of gene expression values from all infected groups (without healthy controls) were correlated by ANOVA with different clinical parameters of patients in the cohort. For virus pathogens, the following groups were used: DenV, ENV_RHVIAV, MPV other_viruses (all other viruses combined). For the age groups the following categories were used: young (0 to 18), adult (18-65), old (>65).

#### Table S11: Hospital admissions


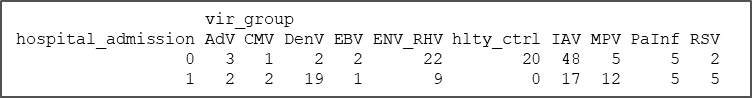


Hospital admissions (0: no hospitalization, 1: hospitalization) for each pathogen group.
