# Supplementary material for: The Host Response to Viral Infections Reveals Common and Virus-Specific Signatures in the Peripheral Blood
Source: Front Immunol. 2021 Oct 27;12:741837. doi: 10.3389/fimmu.2021.741837 (PMC8578928; doi:10.3389/fimmu.2021.741837)
Supplement: Supplementary file 2 [file Table_1.docx]

| **Subject Number** | **Viral Agent** | **Diagnostic Modality** | **Diagnostic Test** |
| --- | --- | --- | --- |
|  | Enterovirus/Rhinovirus | PCR | RVP |
|  | Enterovirus/Rhinovirus | PCR | RVP |
|  | Enterovirus/Rhinovirus | PCR | RVP |
|  | Enterovirus/Rhinovirus | PCR | RVP |
|  | Enterovirus/Rhinovirus | PCR | RVP |
|  | Enterovirus/Rhinovirus | PCR | RVP |
|  | Metapneumovirus | PCR | RVP |
|  | Parainfluenza | PCR | RVP |
|  | Influenza | PCR | RVP |
|  | Influenza | PCR | RVP |
|  | Metapneumovirus | PCR | RVP |
|  | Enterovirus/Rhinovirus | PCR | RVP |
|  | Parainfluenza | PCR | RVP |
|  | Influenza | PCR | RVP |
|  | Respiratory Syncytial Virus | PCR | RVP |
|  | Enterovirus/Rhinovirus | PCR | RVP |
|  | Adenovirus | PCR | RVP |
|  | Influenza | PCR | RVP |
|  | Enterovirus/Rhinovirus | PCR | RVP |
|  | Influenza | PCR | RVP |
|  | Metapneumovirus | PCR | RVP |
|  | Respiratory Syncytial Virus | PCR | Clinical Respiratory PCR |
|  | Influenza | PCR | RVP |
|  | Enterovirus/Rhinovirus | PCR | RVP |
|  | Metapneumovirus | PCR | RVP |
|  | Respiratory Syncytial Virus | PCR | Clinical Respiratory PCR |
|  | Adenovirus | PCR | RVP |
|  | Parainfluenza | PCR | RVP |
|  | Respiratory Syncytial Virus | PCR | RVP |
|  | Metapneumovirus | PCR | RVP |
|  | Adenovirus | PCR | RVP |
|  | Metapneumovirus | PCR | RVP |
|  | Enterovirus/Rhinovirus | PCR | RVP |
|  | Enterovirus/Rhinovirus | PCR | RVP |
|  | Enterovirus/Rhinovirus | PCR | RVP |
|  | Enterovirus/Rhinovirus | PCR | RVP |
|  | Enterovirus/Rhinovirus | PCR | RVP |
|  | Enterovirus/Rhinovirus | PCR | Clinical Respiratory PCR |
|  | Respiratory Syncytial Virus | PCR | RVP |
|  | Respiratory Syncytial Virus | PCR | RVP |
|  | Metapneumovirus | PCR | RVP |
|  | Influenza | PCR | RVP |
|  | Enterovirus/Rhinovirus | PCR | RVP |
|  | Epstein Barr Virus | PCR | Serum EBV PCR |
|  | Herpesvirus | PCR | RVP |
|  | Influenza | PCR | RVP |
|  | Metapneumovirus | PCR | ResPlexTM II |
|  | Herpesvirus | PCR | ResPlexTM II |
|  | Metapneumovirus | PCR | ResPlexTM II |
|  | Parainfluenza | PCR | ResPlexTM II |
|  | Parainfluenza | PCR | ResPlexTM II |
|  | Herpesvirus | PCR | ResPlexTM II |
|  | Influenza | PCR | ResPlexTM II |
|  | Influenza | PCR | ResPlexTM II |
|  | Influenza | PCR | ResPlexTM II |
|  | Influenza | PCR | ResPlexTM II |
|  | Influenza | PCR | ResPlexTM II |
|  | Influenza | PCR | ResPlexTM II |
|  | Influenza | PCR | ResPlexTM II |
|  | Metapneumovirus | PCR | ResPlexTM II |
|  | Metapneumovirus | PCR | ResPlexTM II |
|  | Influenza | PCR | ResPlexTM II |
|  | Epstein Barr Virus | PCR | ResPlexTM II |
|  | Herpesvirus | PCR | ResPlexTM II |
|  | Dengue virus | PCR | ResPlexTM II |
|  | Influenza | PCR | ResPlexTM II |
|  | Influenza | PCR | ResPlexTM II |
|  | Influenza | PCR | ResPlexTM II |
|  | Influenza | PCR | ResPlexTM II |
|  | Influenza | PCR | ResPlexTM II |
|  | Influenza | PCR | ResPlexTM II |
|  | Influenza | PCR | ResPlexTM II |
|  | Influenza | PCR | ResPlexTM II |
|  | Influenza | PCR | ResPlexTM II |
|  | Influenza | PCR | ResPlexTM II |
|  | Influenza | PCR | ResPlexTM II |
|  | Influenza | PCR | ResPlexTM II |
|  | Influenza | PCR | ResPlexTM II |
|  | Enterovirus/Rhinovirus | PCR | ResPlexTM II |
|  | Influenza | PCR | Clinical Respiratory PCR |
|  | Enterovirus/Rhinovirus | PCR | ResPlexTM II |
|  | Enterovirus/Rhinovirus | PCR | ResPlexTM II |
|  | Enterovirus/Rhinovirus | PCR | ResPlexTM II |
|  | Enterovirus/Rhinovirus | PCR | ResPlexTM II |
|  | Enterovirus/Rhinovirus | PCR | ResPlexTM II |
|  | Cytomegalovirus | PCR | Clinical CMV DNA PCR |
|  | Enterovirus/Rhinovirus | PCR | ResPlexTM II |
|  | Metapneumovirus | PCR | ResPlexTM II |
|  | Cytomegalovirus | PCR | Clinical CMV DNA PCR |
|  | Influenza | PCR | Clinical Respiratory PCR |
|  | Adenovirus | PCR | ResPlexTM II |
|  | Cytomegalovirus | PCR | Clinical CMV DNA PCR |
|  | Influenza | PCR | Clinical Respiratory PCR |
|  | Influenza | PCR | ResPlexTM II |
|  | Influenza | PCR | ResPlexTM II |
|  | Influenza | PCR | ResPlexTM II |
|  | Metapneumovirus | PCR | ResPlexTM II |
|  | Influenza | PCR | ResPlexTM II |
|  | Metapneumovirus | PCR | ResPlexTM II |
|  | Influenza | PCR | ResPlexTM II |
|  | Influenza | PCR | ResPlexTM II |
|  | Metapneumovirus | PCR | ResPlexTM II |
|  | Influenza | PCR | ResPlexTM II |
|  | Epstein Barr Virus | PCR | Clinical Serum EBV PCR |
|  | Adenovirus | PCR | ResPlexTM II |
|  | Respiratory Syncytial Virus | PCR | ResPlexTM II |
|  | Metapneumovirus | PCR | ResPlexTM II |
|  | Influenza | PCR | ResPlexTM II |
|  | Influenza | PCR | ResPlexTM II |
|  | Parainfluenza | PCR | Clinical Respiratory PCR |
|  | Parainfluenza | PCR | ResPlexTM II |
|  | Herpesvirus | PCR | Clinical HSV PCR |
|  | Dengue virus | Antibody | Seroconversion |
|  | Metapneumovirus | PCR | RVP |
|  | Influenza | PCR | ResPlexTM II |
|  | Enterovirus/Rhinovirus | PCR | ResPlexTM II |
|  | Influenza | PCR | ResPlexTM II |
|  | Enterovirus/Rhinovirus | PCR | ResPlexTM II |
|  | Influenza | PCR | ResPlexTM II |
|  | Influenza | PCR | ResPlexTM II |
|  | Influenza | PCR | ResPlexTM II |
|  | Influenza | PCR | ResPlexTM II |
|  | Influenza | PCR | ResPlexTM II |
|  | Influenza | PCR | ResPlexTM II |
|  | Influenza | PCR | ResPlexTM II |
|  | Influenza | PCR | ResPlexTM II |
|  | Influenza | PCR | ResPlexTM II |
|  | Influenza | PCR | ResPlexTM II |
|  | Influenza | PCR | ResPlexTM II |
|  | Influenza | PCR | ResPlexTM II |
|  | Influenza | PCR | ResPlexTM II |
|  | Influenza | PCR | ResPlexTM II |
|  | Influenza | PCR | ResPlexTM II |
|  | Parainfluenza | PCR | ResPlexTM II |
|  | Enterovirus/Rhinovirus | PCR | ResPlexTM II |
|  | Parainfluenza | PCR | ResPlexTM II |
|  | Influenza | PCR | NxTAG RPP RUO |
|  | Dengue virus | Antibody | Seroconversion |
|  | Dengue virus | Antibody | Seroconversion |
|  | Dengue virus | Antibody | Seroconversion |
|  | Enterovirus/Rhinovirus | PCR | NxTAG RPP RUO |
|  | Dengue virus | Antibody | Seroconversion |
|  | Dengue virus | Antibody | Seroconversion |
|  | Dengue virus | PCR | Dengue PCR |
|  | Dengue virus | PCR | Dengue PCR |
|  | Dengue virus | PCR | Dengue PCR |
|  | Dengue virus | PCR | Dengue PCR |
|  | Dengue virus | PCR | Dengue PCR |
|  | Dengue virus | Antibody | Seroconversion |
|  | Parainfluenza | PCR | NxTAG RPP RUO |
|  | Influenza | PCR | NxTAG RPP RUO |
|  | Dengue virus | PCR | Dengue PCR |
|  | Influenza | PCR | NxTAG RPP RUO |
|  | Enterovirus/Rhinovirus | PCR | NxTAG RPP RUO |
|  | Influenza | PCR | NxTAG RPP RUO |
|  | Influenza | PCR | NxTAG RPP RUO |
|  | Influenza | PCR | NxTAG RPP RUO |
|  | Dengue virus | Antibody | Seroconversion |
|  | Dengue virus | Antibody | Seroconversion |
|  | Dengue virus | Antibody | Seroconversion |
|  | Dengue virus | Antibody | Seroconversion |
|  | Dengue virus | PCR | Dengue PCR |
|  | Dengue virus | Antibody | Seroconversion |
|  | Dengue virus | Antibody | Seroconversion |
|  | Influenza | PCR | NxTAG RPP RUO |
|  | Enterovirus/Rhinovirus | PCR | NxTAG RPP RUO |
|  | Enterovirus/Rhinovirus | PCR | NxTAG RPP RUO |
